# Topographically guided hierarchical mineralization

**DOI:** 10.1016/j.mtbio.2021.100119

**Published:** 2021-06-09

**Authors:** X. Deng, A. Hasan, S. Elsharkawy, E. Tejeda-Montes, N.V. Tarakina, G. Greco, E. Nikulina, J.M. Stormonth-Darling, N. Convery, J.C. Rodriguez-Cabello, A. Boyde, N. Gadegaard, N.M. Pugno, M. Al-Jawad, A. Mata

**Affiliations:** a School of Engineering and Materials Science, Queen Mary University of London, London, E1 4NS, UK; b Institute of Bioengineering, Queen Mary University of London, London, E1 4NS, UK; c School of Pharmacy, University of Nottingham, Nottingham, NG7 2RD, UK; d Department of Chemical and Environmental Engineering, University of Nottingham, Nottingham, NG7 2RD, UK; e Biodiscovery Institute, University of Nottingham, Nottingham, NG7 2RD, UK; f Faculty of Dentistry, Oral & Craniofacial Sciences, King's College London, London, SE1 9RT, UK; g Mabxience, Insud Pharma, León, Spain; h Max Planck Institute of Colloids and Interfaces, Potsdam-Golm Science Park, Am Mühlenberg 1 OT Golm, Potsdam, 14476, Germany; i Laboratory of Bio-Inspired, Bionic, Nano, Meta, Materials & Mechanics, Department of Civil, Environmental and Mechanical Engineering, University of Trento, Trento, 38122, Italy; j CIC nanoGUNE BRTA, Tolosa Hiribidea, 76, Donostia – San Sebastian, E-20018, Spain; k James Watt School of Engineering, University of Glasgow, Glasgow, G12 8QQ, UK; l BIOFORGE Group, University of Valladolid, CIBER-BBN, Valladolid, Spain; m Oral Bioengineering, Queen Mary University of London, London, E1 4NS, UK; n School of Dentistry, University of Leeds, Leeds, LS2 9JT, UK

**Keywords:** Fluorapatite, Protein-based biomineralization, Hierarchical mineralization, Elastin-like recombinamer, Bone, Dental enamel, Crystallization, Surface topographies

## Abstract

Material platforms based on interaction between organic and inorganic phases offer enormous potential to develop materials that can recreate the structural and functional properties of biological systems. However, the capability of organic-mediated mineralizing strategies to guide mineralization with spatial control remains a major limitation. Here, we report on the integration of a protein-based mineralizing matrix with surface topographies to grow spatially guided mineralized structures. We reveal how well-defined geometrical spaces defined within the organic matrix by the surface topographies can trigger subtle changes in single nanocrystal co-alignment, which are then translated to drastic changes in mineralization at the microscale and macroscale. Furthermore, through systematic modifications of the surface topographies, we demonstrate the possibility of selectively guiding the growth of hierarchically mineralized structures. We foresee that the capacity to direct the anisotropic growth of such structures would have important implications in the design of biomineralizing synthetic materials to repair or regenerate hard tissues.

## Graphical abstract

**Figure:**
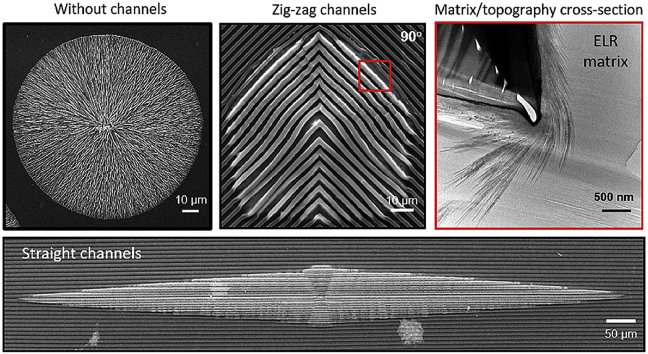

## 1 Introduction

Mineralized tissues in nature, such as nacre, bone, and dental enamel, exhibit a rich spectrum of remarkable functionalities as a result of their anisotropic structures, which are spatially organized at different length scales [1,2]. Enamel, for example, is composed of hydroxyapatite (HAP) nanocrystals that bundle together to form either thick prisms or differently oriented interprismatic regions [3,4]. This structural anisotropy, resulting from an intricate spatial organization of nanocrystals, provides remarkable mechanical properties [5] and chemical stability [6]. In spite of the realization of the potential of these structures, there remains an unmet need to recreate such functional architectures [7]. Toward this goal, a variety of innovative mineralizing material platforms based on organic matrices are emerging.

The supramolecular organization of organic matrices plays a key role in regulating the interaction between organic and inorganic phases that control mineral nucleation and growth [8]. Inspired by the role of amelogenin in enamel development, self-assembled amelogenin nanoribbons [9], synthetic amelogenin-mimetic peptide coatings [10], and phase transited lysozyme films comprising N-terminal amelogenin domain and synthetic peptides [11] have been shown to control apatite nucleation and template crystallites with preferential growth along the c-axis and integrating firmly to etched enamel tissue. The role of collagen has also been explored. For example, dentin phosphophoryn–inspired phosphopeptides with (SSD)_3_ motifs were developed to induce biomineralization with spatially organized intrafibrillar apatitic crystallites nucleating from collagen fibrils [12]. Furthermore, self-assembling elastin-like recombinamer (ELR) fibers have been reported to undergo collagen-like intrafibrillar mineralization via spatially confined ELR β-spiral structures [13]. Other examples also based on supramolecular matrix-guided biomineralization have been explored [[14], [15], [16], [17]]. Despite these advances, however, key challenges remain such as the capacity to control kinetics of crystal precipitation, guide the orientation of crystal growth, and ultimately generate rationally designed hierarchical macrostructures.

The capacity to guide mineralization with spatial control is critical, as the intricate organization of individual nanocrystals and their arrangement at higher size scales determine the mechanical properties of the resulting material [18]. In this effort, the supramolecular organic matrices play a key role [19]. For example, amelogenin-chitosan hydrogel matrices have been used to stabilize and guide the aggregation of calcium phosphate (CaP) clusters into bundles of co-aligned high aspect ratio crystals [20]. These crystals integrate and grow on an acid-etched enamel substrate via a cluster growth process. In another example, Wang et al. reported biomimetic remineralization of demineralized enamel surfaces using an organic matrix and glycine-guided HAP nanoparticles to form ordered and oriented needle-like elongated apatite crystals through a non-classical crystallization process [21]. Recently, we demonstrated how ELR membranes with tunable levels of conformational order and disorder can nucleate well-defined HAP nanocrystals and guide their growth into hierarchical macrostructures [22]. However, this process does not provide spatial control, which would have important implications in the design of more complex and functional mineralizing materials.

The use of volumetrically [23] and topographically [24] confined environments has been explored to guide crystal precipitation and formation with spatiotemporal control [[25], [26], [27]]. For example, calcium carbonate precipitation and co-oriented calcite nanocrystals have been grown within confined nanoscale volumes inside collagen nanofibrils [28]. We have previously demonstrated that a volumetrically confined ELR matrix [22] is able to generate hierarchical biomineralization, whereas the same ELRs presented as unconfined surface coatings did not [29]. Others have also reported on the precipitation and transformation of CaP ions during biomimetic apatite formation under confined volumes of cross-linked gelatin at both the nanoscale [23] and microscale [30]. In addition, topographically confined regions of nanoporous [31] and microporous [24] structures exposed to calcium carbonate precipitation have been reported to affect ion diffusion and control growth kinetics of crystal formation. Therefore, taking advantage of patterned templates with well-defined geometrical topographies offers an attractive method to form mineralized macrostructures with spatial control and anisotropic organization. For example, patterned macroporous polymeric templates have been used to precipitate calcium carbonate and trigger diffusion-mediated growth of spatially guided calcite crystals exhibiting porous and sponge-like three-dimensional (3D) morphologies [32]. Nonetheless, despite great interest in mineralizing strategies based on organic matrices [33,34], there is a limited capacity to design such organic materials capable of guiding the orientation and alignment of crystallites within a hierarchical mineralized structure with spatial control. Furthermore, the identification of parameters that play a role in the growth of such structures would shed light on underlying mineralization mechanisms and open opportunities for the synthesis of advanced mineralized materials.

In this study, we investigate the use of precise surface microtopographies on ELR membranes to grow apatite nanocrystals with spatial control. Our approach offers a novel platform to integrate the volumetrically and topographically confined organic matrix to spatially guide mineralization. Our results demonstrate the capacity to use surface topographies to affect and guide mineralization and the direction of crystal growth both within the bulk and on the surface of the matrix. We envisage the ability to spatially directing the anisotropic growth of such mineralized structures would open promising avenues for the repair or regeneration of hard tissues such as dental enamel and bone.

## 2 Experimental section

### 2.1 Chrome mask photolithography fabrication

A chrome photomask was designed in Tanner L-Edit and fabricated by Micro Lithography Services Ltd. (UK). Briefly, a 4-inch silicon wafer (Pi-kem, UK) was washed sequentially in acetone, methanol, and isopropyl-alcohol (IPA) for 5 min each in an ultrasonic bath and dried under nitrogen. The washed wafers were further dehydrated for 2 h at 180°C and surface treated with oxygen plasma at 100 W for 2 min. Before spinning the S1818 resit, the wafers were coated with hexamethyldisilazane primer, both at 4000 rpm for 30 s. The resist was then soft baked for 2 min at 115°C on a vacuum hotplate. The resist was patterned through the photomask on an MA6 mask aligner (Süss MicroTec, Germany), exposure dose 42 mJ/cm^2^ before development in MF-319 developer for 75 s with gentle agitation. Excess developer was washed off in reverse osmosis water and dried under nitrogen before the wafer and pattered resit were subject to oxygen plasma at 80 W for 30 s to descum. Next, the patterned wafer was mounted to a carrier wafer (Cool Grease; AITechnology, USA) before etched in an STS-ICP etch tool (Surface Technology Systems, UK‘ SF_6_/C_4_F_8_ = 40/50 sccm, coil/platen = 600/12 W, 10 mTorr, 20°C for 6 min and 30 s) to achieve an etch depth of 5 μm. Finally, the resist was stripped overnight in SVC-14 at 50°C, before the wafer was washed in acetone, methanol, IPA for 5 min each, and dried under nitrogen completely. Polydimethylsiloxane (PDMS) masters were prepared using the method described previously [35]. Briefly, components of the PDMS kit (Sylgard 184, Dow Corning, Midland, MI), which included PDMS base and curing agent, were mixed in a ratio of 10:1 and degassed for 7 min followed by pouring on top of the patterned Si master. After additional 12 min of degassing, PDMS on Si master was cured at 65°C for 3 h and at room temperature (~25 °C) for 21 h.

### 2.2 ELR membrane fabrication

**Fig. 1:**
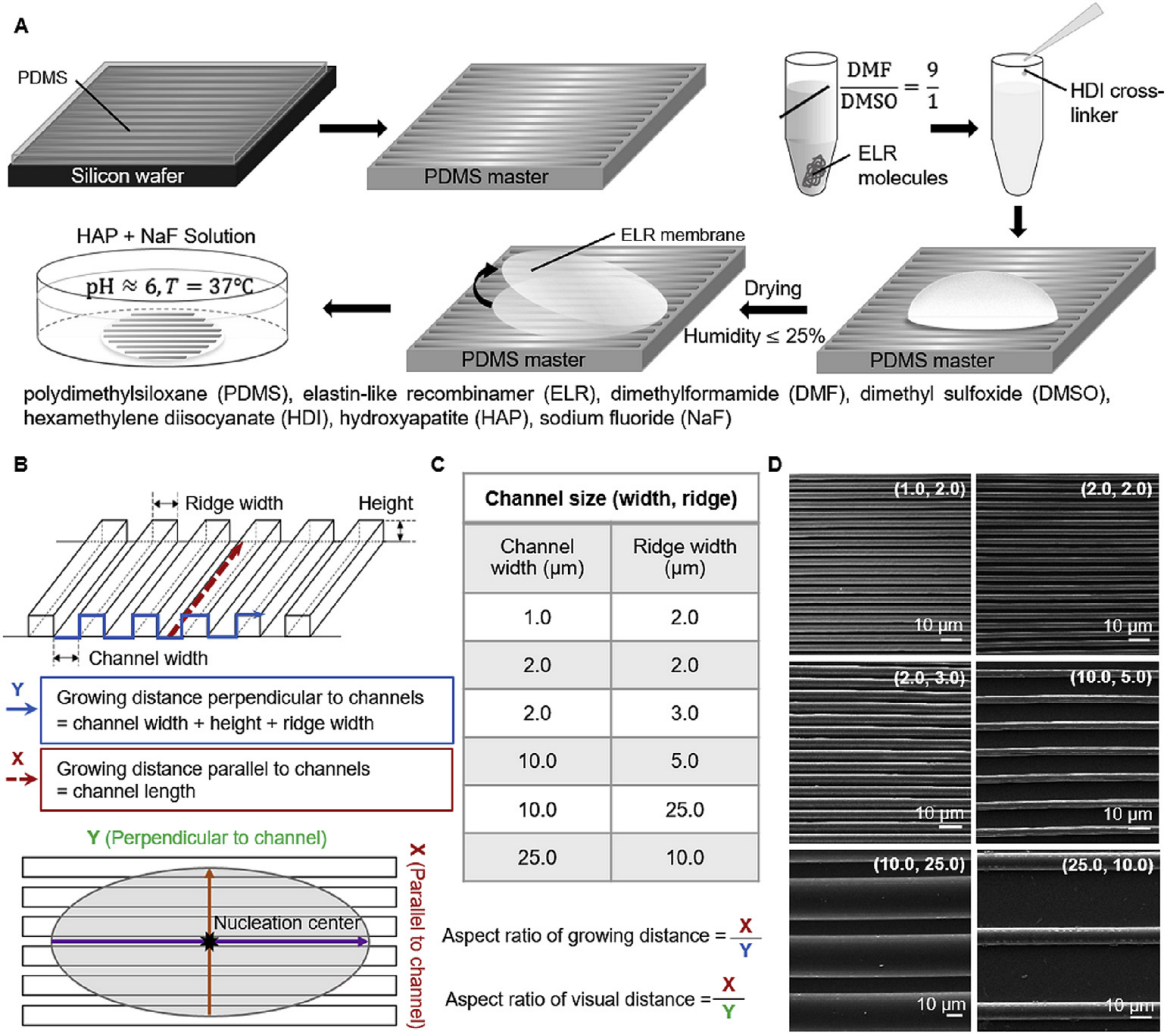
**Fabrication of topographically patterned surfaces.** (A) Schematic illustration of the fabrication of PDMS master and topographically patterned ELR membranes by soft lithography followed by membrane mineralization. (B) 3D representation of microchannel patterns depicting channel width and ridge width and the aspect ratio calculation to characterize the mineralized structures based on the distance covered in the direction parallel (X) and perpendicular (Y) to the channels. (C) Table with the different dimensions of the microchannel surface topographies. (D) SEM images of the patterned ELR membranes before mineralization specifying the respective channel dimensions.

ELR membranes were fabricated using the procedure described previously by our group [22]. Briefly, ELR molecules (Technical Proteins Nanobiotechnology, Valladolid, Spain) were dissolved in solvent mixture of anhydrous dimethyformamide (Sigma-Aldrich, UK) and dimethyl sulfoxide (Sigma-Aldrich, UK) at a ratio of 9/1 at room temperature inside a polymer glovebox (BELLE Technology, UK) under controlled humidity (<20%). The resultant solution was then cross-linked with hexamethylene diisocyanate (HDI; Sigma-Aldrich, UK), drop casted, and left for drying overnight on top of the smooth and topographically patterned PDMS masters (Fig. 1A). Dried ELR membranes were carefully peeled off from the PDMS masters and washed several times with dimethyl sulfoxide (DMSO) followed by de-ionized water to get rid of excess HDI and finally stored at 4°C until use.

### 2.3 Mineralization experiment

Mineralizing solution was prepared by dissolving HAP powder (2 mM) and sodium fluoride (2 mM) in de-ionized water under continuous stirring. The powder was completely dissolved by adding nitric acid (69%) dropwise into the solution until it becomes clear. Ammonium hydroxide solution (30%) was later added dropwise into the clear solution to readjust the pH value to 6.0 [36]. Fabricated smooth and topographically patterned membranes were then dipped in the mineralization solution and incubated for 8 days at 37°C inside a temperature-controlled incubator (LTE Scientific, Oldham, UK).

### 2.4 Scanning electron microscopy and energy-dispersive X-ray spectroscopy

Mineralized ELR membranes were dried under nitrogen and mounted on aluminum stubs using self-adhesive carbon tape followed by coating with conductive material (gold or carbon) via auto-sputter coating machine and observed using and FEI Inspect F (Hillsboro, OR, USA). The surface topographies of non-mineralized membranes and morphology of the mineralized microstructures were investigated using scanning electron microscopy (SEM; Hillsboro, OR, USA), and the chemistry elemental analysis was performed using INCA software through the spectra mapping of interested areas collected via energy-dispersive X-ray spectroscopy detector (INCA x-act; Oxford Instruments) at an accelerating voltage of 10 kV [37].

### 2.5 Focused ion beam-SEM (FIB-SEM)

Transmission electron microscopy (TEM) specimens were prepared using an FEI Quanta 3D ESEM (Hillsboro, OR, USA). Mineralized structure was milled by focused gallium beam of which the parameters were set to 30 kV and 5 nA. Then the stairs cut technique followed by an in situ lift-out operation and polishing was applied afterward. The cross-section cleaning was applied with 30 kV ion beam, and the current went down from 100 pA to 28 pA for both sides of the specimen at 2° incidence angle until the lamella was around 150 nm in thickness. The final polishing was done with the parameter of 5 kV, 16 pA, and 1° incidence angle to thin the lamella below 100 nm [38].

### 2.6 Transmission electron microscopy

TEM study was performed using a JEOL 2010 operated at 120 KV, and a double Cs corrected JEOL JEM-ARM200F (S)TEM operated at 80 kV, equipped with a LaB6 and a cold-field emission gun, correspondingly. All measurements were made on focused ion beam (FIB)-SEM lamellas prepared from different samples. The obtained high-resolution images and selected area electron diffraction (SAED) patterns were analyzed using the Gatan Microscopy Suite (GMS 3) software. Interplanar distances calculated from SAED patterns were compared with the standard powder diffraction file PDF2 database (ICDD, USA, release 2009) and were found to be in correspondence with X-ray diffraction (XRD) measurements.

### 2.7 X-ray diffraction

An X'Pert Pro X-ray diffractometer (PANalytical, B.V., Almelo, the Netherlands) was used to analyze the phase composition of the mineralized structures at room temperature. Instrument was operated with flat plate θ/θ geometry and Ni-filtered Cu-Kα radiation at 45 kV and 40 mA (Kα1 = 1.54059 Å, Kα1 = 1.54442 Å) [39]. The 2θ values were recorded from 5° to 70° with a step size 0.0334°, and data were obtained via PANalytical X'Celerator solid-state real-time multiple strip detector continuously with an equivalent step time of 1600 s. X'pert high score (3.0e) with the PDF4 database (ICDD, USA, release 2014) was used for comparison.

### 2.8 Fourier-transform infrared spectroscopy

Fourier-transform infrared (FTIR) spectroscopy analysis of ELR membranes before and after mineralization was performed using FTIR Spectrum GX (PerkinElmer; Waltham, MA, USA). ELR membranes were placed over the infrared window and recorded 128 scans on average at a resolution of 4 per cm in the wavenumber range of 4000 per cm to 450 per cm in respect to the percentage of absorbance and the percentage of transmittance for organic and inorganic samples, respectively [22]. The spectrum data were analyzed by OMNIC software, and the original data values were dealt with Origin software to make the final spectrum curve.

### 2.9 Nanoindentation

Mineralized membranes with channel (0, 0) and channel (2, 2) were glued using Loctite Super Attack glue and left for drying for 24 h. Young's modulus (*E*) measurements were performed with instrument iNano Nanoindenter (Nanomechanics Inc.) with the sensitivity of 3 nN for the load and 0.001 nm for the displacement. The membranes were analyzed with single indentations and mapping procedure (Nanoblitz 3d [Nanomechanics Inc.] using method reported previously [22,40]. A Berkovich tip was mounted in the machine and used to perform the indentation with the 1–2 points per microns, indentation depth ranging between 30 and 70 nm, and the load ranging between 0.01 and 0.05 mN at 20°C and 30–40% relative humidity. The mechanical properties were computed using the well-established method described by Oliver and Pharr [41].

## 3 Results and discussions

### 3.1 Rationale of design

**Fig. 2:**
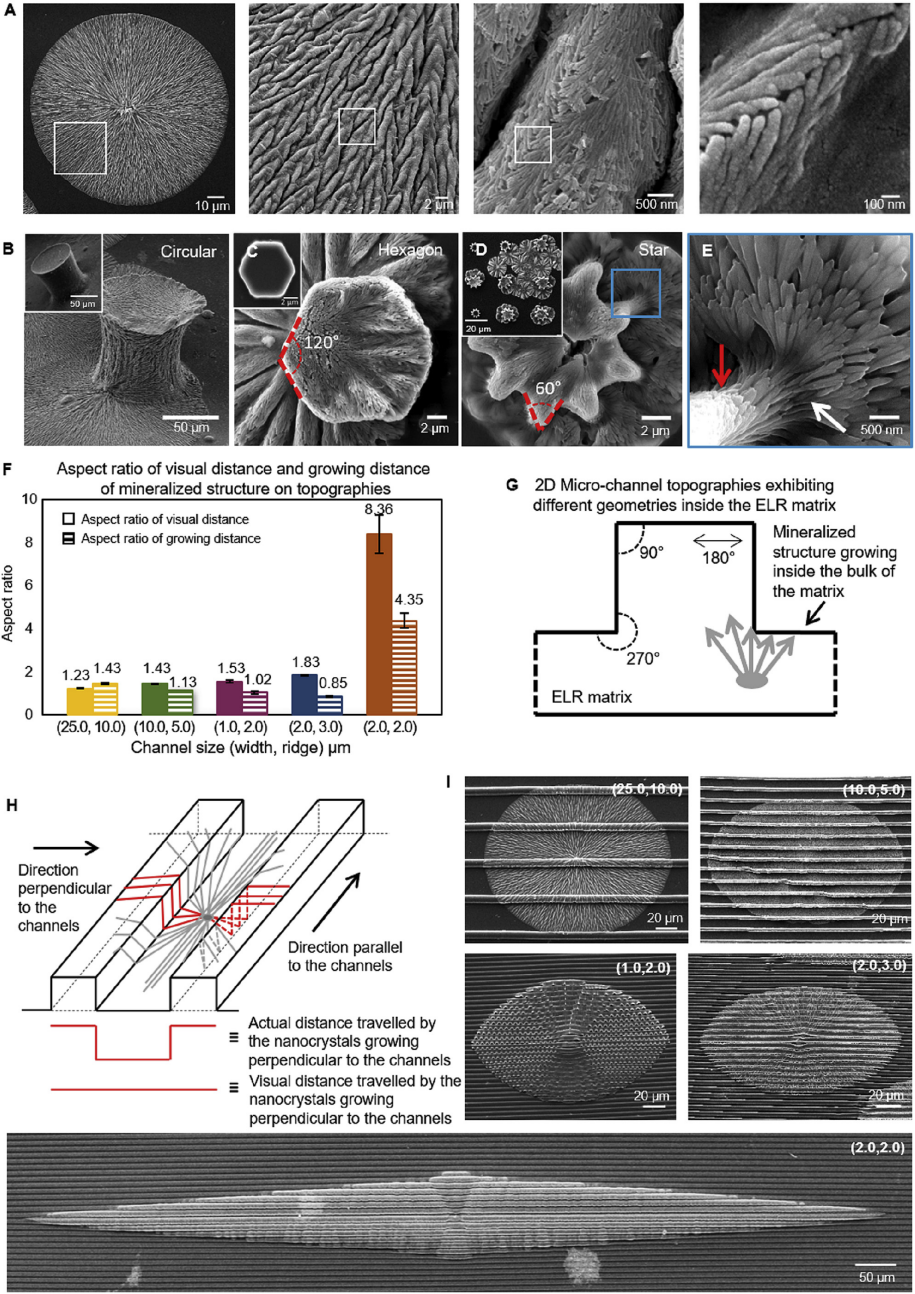
**Mineralization of ELR membranes.** (A) SEM images of a hierarchical mineralized structure grown on a smooth ELR membrane. Mineralized structures grown on the top of ELR membranes with (**B)** circular-, (**C)** hexagon-, and (**D)** star-shaped post topographies. Insets in B and C show non-mineralized circular and hexagonal topographies. Panel **E** highlighting the different co-alignment between apatite nanocrystals growing on the vertical side of the post (red arrow) and at the junction between the vertical side of the post and the horizontal space between them (white arrow). (**F)** Graph summarizing the relation between channel dimension and aspect ratio of the mineralized structures grown on patterned ELR membranes. Solid bars correspond to the aspect ratio of visual distance, and striped bars correspond to aspect ratio of growing distance. (**G)** Cross-section of a microchannel topography highlighting different geometrical spaces within the ELR matrix defined by the topographies including 90°, 180°, and 270° angles. (**H)** Illustration depicting a mineralized structure with actual and virtual distances traveled by the nanocrystals. (**I)** SEM images of spherulitic mineralized structures growing on different microchannel dimensions exhibiting different levels of elongation.

**Fig. 3:**
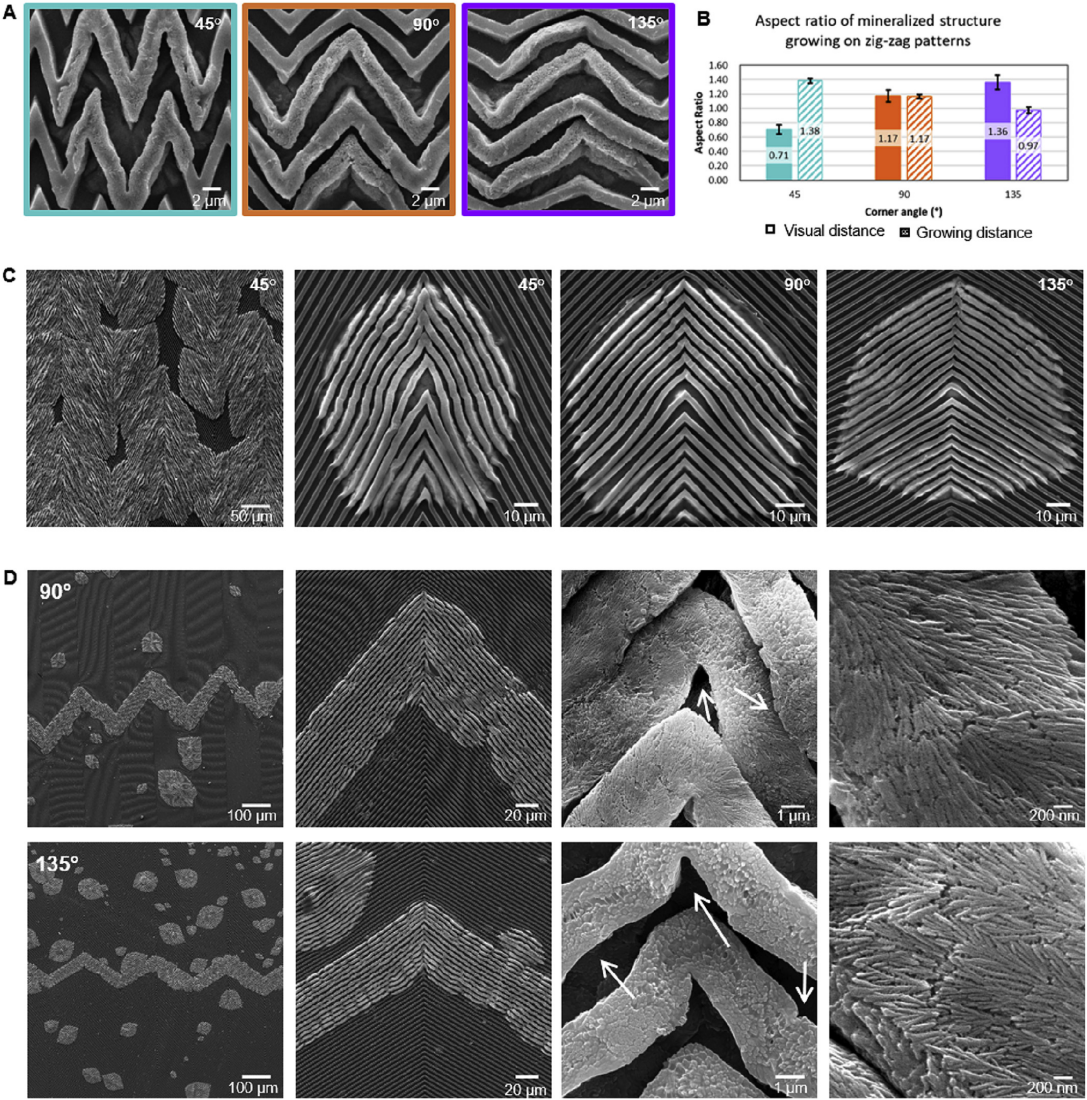
**Characterization of mineralized membranes with zig-zag microchannels.(A**, **C)** SEM images of mineralized structures grown on ELR membranes with zig-zag Channel (2, 2) patterns aligned at 45°, 90°, and 135° corner angles. (**B)** Graph depicting the relation between the aspect ratio of the mineralized structures and the corner angles of the zig-zag patterns. Solid bars correspond to aspect ratio of visual distance and striped bars correspond to aspect ratio of growing distance. (**D)** SEM images of the mineralized structures growing along the channel directions and guided by the zig-zag patterns (with 90° and 135° corner angles) over a millimeter length scale. Minor distortions in the channel geometry appeared on zig-zag patterns (highlighted by arrows) in the presence of the mineralized structures, suggesting the presence of stresses being generated by the growing nanocrystals.

Our approach combines a protein-based matrix capable of triggering hierarchical mineralization with well-defined surface topographies. The protein matrix was fabricated using ELR molecules (Fig. 1), which are recombinant elastin-mimicking polypeptides consisting of hydrophobic domains (VPGIG), positively charged domains (VPGKG) with intermittent lysine (K) segments for cross-linking, and a statherin-derived mineralizing sequence DDDEEKFLRRIGRFG (SN_A_15) [29,42]. We have recently demonstrated that by modulating ELR order and disorder, it is possible to grow high aspect ratio 50 nm thick apatite nanocrystals that are hierarchically organized in ~5 μm diameter microbundles and spherulitic mineralized structures of up to 1 mm in diameter (Fig. 2A) [22]. We hypothesized that by creating well-defined topographies on the surface of this ELR matrix, geometrically confined ELR volumes would reproducibly affect the growth of the apatite nanocrystals and microbundles. Therefore, topographical patterns comprising posts with star or hexagonal shapes and channels with straight (Fig. 1D) or zig-zag (Fig. 3A and Supplementary Fig. S1) profiles were fabricated exhibiting different sizes, angles, and frequencies ranging between 1 μm and 25 μm in size (Fig. 1C and D) and angles between 45° and 270° (Fig. 2G). The channels are expressed as ‘Channel (channel width and ridge width)’ throughout the manuscript; for example, ‘Channel (2, 2)’ indicates channel width and ridge width of 2 μm each. By systematically changing the channel dimensions and geometries, we investigated the effect of confined ELR volumes on guiding the growth of the mineralized macrostructures.

### 3.2 Membrane fabrication, characterization, and mineralization

#### 3.2.1 Fabrication of membranes with surface topographies

We first fabricated topographically patterned silicon wafers via photolithography followed by reactive ion etching [43]. These patterned silicon wafers were then used to cast PDMS masters [35], and themselves used to cast ELR membranes via soft lithography at room temperature by cross-linking and drying the ELR solution as previously described [22] but directly on top of the topographically patterned PDMS masters (Fig. 1A). SEM analysis revealed that the patterned ELR membranes did not exhibit any geometrical variations with respect to their PDMS masters (Fig. 1D). However, there were minor defects at the tip of ELR post and channel topographies, which likely occurred during one of the soft lithographic steps.

#### 3.2.2 Mineralization of topographical membranes

On exposure of the smooth ELR membranes (i.e. without micropatterns) to a supersaturated mineralizing solution at pH of 6 and 37°C, apatite nanocrystals nucleated within the bulk of the ELR matrix to form the root of the mineralized structure. Nanocrystals from this root grew along their c-axis to emerge and spread radially on the surface of the ELR membrane as previously reported [22]. When the aligned nanocrystals emerged out of the bulk and onto the membrane surface, they organized into microscopic prisms, which grew together into macroscopic circular mineralized structures (Fig. 2A) [22]. The direction of nanocrystal growth was away from the point they emerged onto the surface (Fig. 2A). Similar nucleation of nanocrystals within the ELR bulk and growth of circular mineralized structures on the ELR membranes comprising post topographies (i.e. circular, hexagonal, and star shaped) were observed (Fig. 2B, C, and 2D). Therefore, it is likely that as in the case of the smooth ELR membranes, ions from the mineralizing solution also diffused inside the bulk of the membranes to trigger nucleation. At the macroscale, these mineralized structures grew on different locations of the ELR membranes irrespective of the geometrical shape of the post topographies and on all surfaces (i.e. between adjacent posts as well as on the vertical and top surface of the posts; Fig. 2C). At the nanoscale, however, the nanocrystals were organized differently depending on the geometrical shape of the post topographies. Major differences were observed particularly in the level of nanocrystal alignment at the base of the posts where the vertical walls meet the horizontal space between posts. In these locations of membranes comprising circular posts, nanocrystals maintained co-alignment but together changed direction by 90° as they grew from the post wall to the space between posts (Supplementary Figs. S2A and S2B) and vice-versa (Supplementary Fig. S2C). However, when the post geometry exhibited angles on the vertical walls such as in the case of hexagonal posts (i.e. 120°), nanocrystal co-alignment changed (Fig. 2C). This effect was particularly pronounced on the star-shaped posts exhibiting 60° angles on the walls (Fig. 2D), which lead to less co-aligned nanocrystals (Fig. 2E, pointed with white arrow). These results suggest that nanocrystals growing on the surface of ELR membranes change their co-alignment depending on the geometrical features they encounter, with sharper topographical features resulting in higher misalignment.

### 3.3 Mineralization and characterization of straight channels

#### 3.3.1 Mineralization results of straight channel

Given these topographical effects on nanocrystal co-alignment, we fabricated ELR membranes exhibiting 4 μm deep microchannels of varying channel width and ridge width from 1 μm to 25 μm in size (Fig. 1D). After exposure to the mineralization solution for 8 days, we again observed effects on the mineralized structures at different size scales. In particular, the morphology of the spherulitic mineralized structures on the surface of the ELR membranes changed from circular to elliptical with varying aspect ratios depending on the dimensions of the microchannels (Fig. 2I). Here, we define the aspect ratio of the mineralized structures as the ratio between the distance of parallel growth (i.e. structure length) to the distance of perpendicular growth (i.e. structure width; Fig. 1B). However, this aspect ratio does not take into account the actual distance traveled by the nanocrystals in the direction perpendicular to the channels. In this direction, the nanocrystals would travel longer distances than those defined by the aspect ratio as they go up and down the channel geometry (Fig. 2H). Nonetheless, despite measuring the aspect ratio taking into account the actual traveled distance, the results demonstrate that the nanocrystals are indeed growing longer along the direction of the channels than they do perpendicular to the channels. Thus, based on the measurement of the distance traveled by the nanocrystals in the direction perpendicular to the channels, we define two types of aspect ratios, including ‘aspect ratio of growing distance’ and ‘aspect ratio of visual distance.’ Aspect ratio of growing distance takes into account the actual distance traveled by nanocrystals as they go up and down the channel geometry in the direction perpendicular to the channel, whereas aspect ratio of visual distance considers the virtual distance traveled by the nanocrystals, as visualized from the top of the mineralized structures (Fig. 2H).

#### 3.3.2 Morphologies of the mineralized structures

The morphology of the mineralized structures changed from circular on smooth membranes (Channel (0, 0)) to oval shape on Channel (1, 2) and elliptical on Channel (2, 2), elongating in the direction of the channels (Fig. 2I). Notably, as the sizes of the channels decrease, the aspect ratio of visual distance tended to increase (from 1.22 ± 0.04 to 1.83 ± 0.03), whereas the aspect ratio of growing distance tended to decrease (from 1.43 ± 0.03 to 0.85 ± 0.04). Interestingly, although limited effects on both types of aspect ratio were observed in Channels (25, 10), (10, 5), (1, 2), and (2, 3); a large change was observed in Channel (2, 2) where the aspect ratio of the mineralized structures increased substantially to 8.36 ± 0.88 for visual distance and 4.35 ± 0.34 for growing distance (Fig. 2F). It has been reported that enhanced diffusion and localization of ions can increase the growth of nanocrystals [44,45]. We hypothesize that this enhanced growth of apatite structures along the channel direction may result from an increased localization of Ca and P ions. This is discussed in detail in the section ‘*Effect of different geometries on crystal co-alignment*’. Overall, these results demonstrate the effective guidance that Channel (2, 2) topographies have on the growth of the mineralized structures.

#### 3.3.3 Hypothesis

To elucidate this effect of the Channel (2, 2) topographies, it is important to take into account the kinetics of growth. The mineralized structures are nucleating within the ELR matrix and growing from the bulk and toward the surface of the ELR membranes [22]. As the growing nanocrystals inside the matrix reach the ELR membrane surface, they encounter the microchannel topographies, which constrain the ELR matrix within confined volumes exhibiting different angles including 90°, 180°, and 270° (Fig. 2G). It is possible that these angles at the surface topography regulate the morphology of the mineralized structures by affecting the growth of the nanocrystals in a similar manner as those observed on the post topographies (Fig. 2B, C, and 2D). This effect at the nanocrystal and microbundle levels may be playing a role in the dramatic elongation exhibited by the mineralized structures on Channel (2, 2), whereas mineralized structures on smooth surfaces (Channel (0, 0)) exhibit symmetrical morphology with no directional growth (Fig. 2A). Based on these observations, we addressed two questions: (1) How robust is this topographical guidance effect? and (2) Can the topographical patterns on the ELR membranes be used to guide the directional growth of the nanocrystals?

### 3.4 Mineralization and characterization of zig-zag channels

#### 3.4.1 Aspect ratio of visual distance on zig-zags

To explore these questions, similar surface topographies as those of Channel (2, 2) were fabricated but in zig-zag patterns with variable corner angles, including 45°, 90°, and 135° (Fig. 3A). As in the smooth ELR membranes and those comprising straight channels, the membranes with zig-zag channel topographies also exhibited the spherulitic mineralized structures emerging onto the surface but with different aspect ratios of visual distance depending on the corner angles. The aspect ratio of visual distance of these structures increased from 1 on smooth surface to 1.23 on 90° corner angle patterns and further to 1.40 on 135° corner angle patterns (Fig. 3B and C). This increase in the aspect ratio of visual distance of the structures with increase in the corner angles indicates that as the zig-zag patterns become more like straight channels, a similar trend of mineralized structure alignment along the direction of the channels appears. However, zig-zag channels with 45° corner angles exhibited mineralized structures with the smallest aspect ratio of visual distance (i.e. 0.77) because of mineralized structures growing and elongating in the direction perpendicular to the channel rather than along the channel direction. These results indicate the possibility of switching the direction of mineralization by 90° depending on the zig-zag corner angle.

#### 3.4.2 Aspect ratio of growing distance on zig-zags

In contrast to the aspect ratio of visual distance, we observed completely opposite behavior of the aspect ratio of growing distance, which decreased with the increase in the corner angles of zig-zag channels. Zig-zag channels with 45° corner angles exhibited the highest aspect ratio of growing distance (i.e. 1.38), which decreased to 1.17 on 90° corner angles and further to 0.97 on 135° corner angles (Fig. 3B). Channel ridges of 45° corner angle zig-zag patterns are closer than those of 90° and 135° corner angles. Therefore, on mineralization, nanocrystals encounter more channel ridges on 45° corner angle zig-zag and thus travel longer distances compared with nanocrystals on 90° and 135° corner angle zig-zags. Notably, channel geometry appeared to be distorted on all zig-zag patterns in the presence of the mineralized structures, which is likely because of stresses generated on the ELR matrix by the growing nanocrystals (as highlighted by arrows in Fig. 3D). Small isotropic structures grew along the channel direction with time to acquire the elongated anisotropic form, as evident in the low magnification image in Fig. 3D. Furthermore, in some instances, multiple mineralized structures grew and appeared to emerge close to each other and together followed the channel geometry, creating millimeter-long mineralized zig-zag patterns (Fig. 3D). These results not only demonstrate the robustness of the topographical guidance in modulating the morphology of the mineralized structures but also indicate the possibility to guide them directionally.

### 3.5 Tuneability of the process

#### 3.5.1 Conformation of directional control using zig-zags

**Fig. 4:**
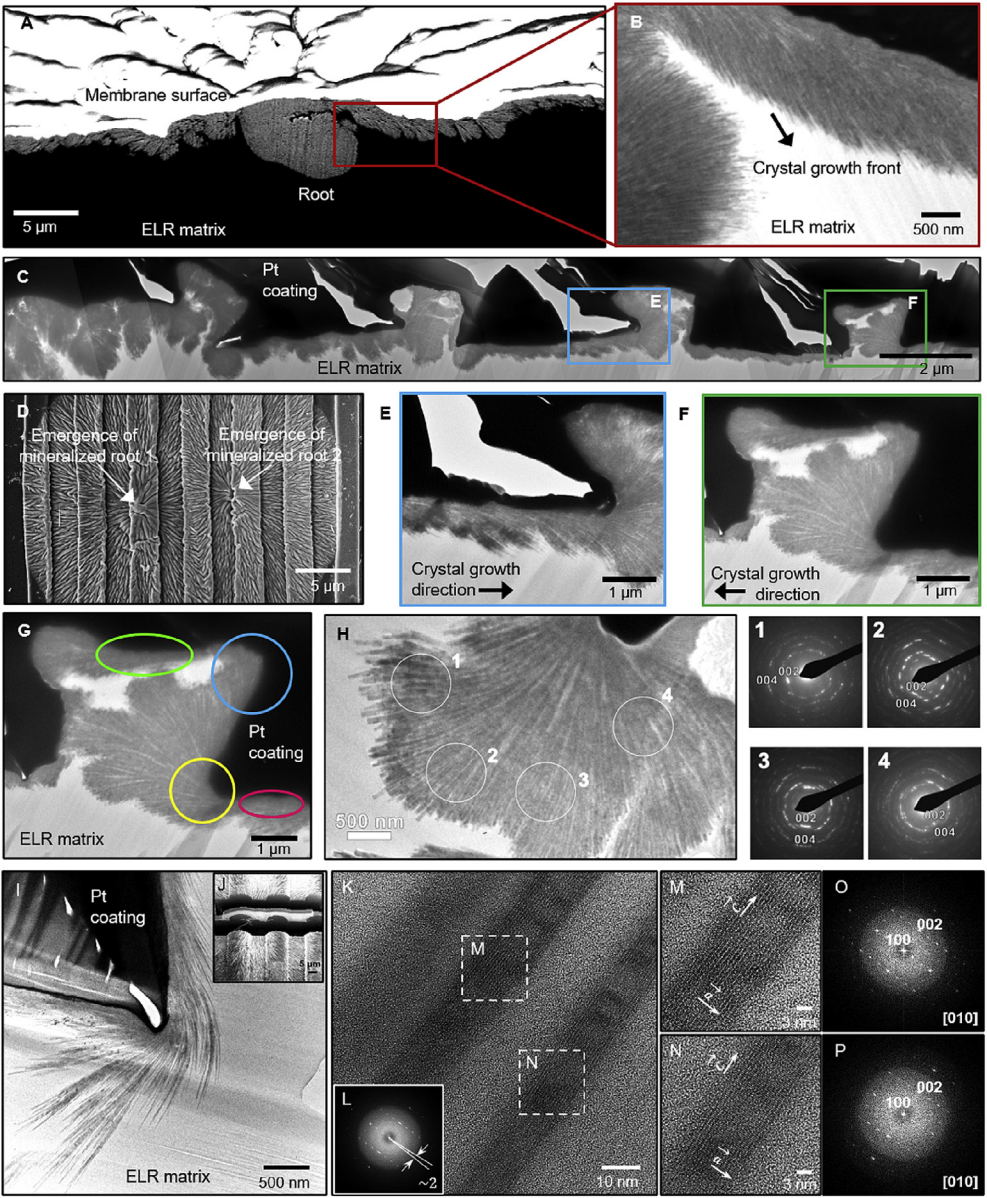
**Bulk characterization.** (A) FIB-SEM and (B) TEM image of the cross-section of a smooth ELR membrane showing the nucleation (root) of the mineralized structure and organization of nanocrystals. (C) TEM image of the cross-section of a Channel (2, 3). (D) SEM image of the two adjacent mineralized structures showing the emergence of mineralized roots. Panels **E and F** show the crystal growth direction within the channel. (**G)** TEM image of the cross-section of Channel (2, 3), illustrating the organization of nanocrystals growing within the ELR membrane at different angles defined by the surface microchannels (pink and green circle at 180° angle, yellow circle at 270° angle, and blue circle at 90° angle). (**H)** HRTEM images and SAED patterns displaying the typical crystallographic characteristics of fluorapatite nanocrystals with flat-ended geometry and growing along the c-axis irrespective of the ELR matrix geometry. (**I)** TEM image of nanocrystals within the ELR matrix of Channel (25, 10). The dark regions in **C, G,** and **I** are because of platinum (Pt) coating on the mineralized structures, which is used to prevent sample damage during FIB milling. (**J)** FIB-SEM image showing cross-sectional lamella preparation via FIB milling. (**K, M, and N)** HRTEM image of a single fluorapatite nanocrystal showing the growth orientation and crystal lattice. (**L, O, and P)** Fast Fourier transform (FFT) diffraction pattern of fluorapatite nanocrystals exhibiting a 2° co-alignment angle.

Our approach offers the possibility of integrating the supramolecular architecture provided by the spherulitic organization of the organic ELR matrix with the microscale-confined architecture provided by the surface topographies to spatially control mineralization. Surface Channel (2, 2) permitted apatite nucleation from the bulk of the ELR membrane but guided the growth of mineralized structures with high aspect ratio of visual distance on the surface (Fig. 2I). Furthermore, by changing the direction of these channels with zig-zag patterns, we further demonstrated that topographies were able to guide the growth of nanocrystals on the surface (Fig. 3A). In addition, the corner angles of these zig-zag patterns affected the mineralization direction (Fig. 3C). These observations confirm the possibility of using surface topographies to guide the growth of the hierarchically organized mineralized structures. However, metallic surfaces with microchannel topographies have been previously reported to not necessarily regulate the directional growth of apatite nanocrystals [46,47]. The effect of confinement on mineralization has been previously reported in both volumetric confinement in ELR [22] and gelatin [30] matrices as well as topographical confinement in nanoporous [31] and microporous [24] structures. In this study, we integrate both volumetric and topographical confinement using the ELR matrix. The topographical effects reported here emerge from both differences in channel size (ranging between 1 and 25 μm) and angles (i.e. 90°, 180°, and 270°) formed by the microchannels within the ELR matrix (Fig. 4G). We have taken advantage of the ordered and hierarchical nature of the mineralization process (i.e. ~50 nm crystals growing into 5 μm prisms and these into structures 100s of μm in diameter; Fig. 2A) to investigate the effects on nanocrystal growth at multiple size scales under confined conditions. To the best of our knowledge, this is the first report that highlights the tuneability of an organic matrix to exhibit precise control on directionally guided growth of hierarchically organized fluorapatite mineralized structures over millimeter length scales.

### 3.6 Characterization of the crystallographic organization

#### 3.6.1 Physical characterization of the bulk

To investigate the underlying mechanism behind this guided mineralization process, we first characterized the crystallographic organization and orientation of the apatite nanocrystals inside the bulk of the ELR matrix. Ultrathin lamellae from mineralized structures grown on smooth membranes (Fig. 4A) and straight Channel (2, 3) (Fig. 4C) were milled out using a FIB and analyzed under a TEM (Fig. 4B and C, respectively). These results revealed the nucleation (i.e. root) of the mineralized structure inside the ELR matrix with the nanocrystals growing along their c-axis and emerging on the surface of the ELR membrane. The growth front of the nanocrystals corresponding to the c-axis grew on the surface of the membrane but was directed toward the bulk of the ELR matrix, as pointed by the arrow in Fig. 4B. Furthermore, these results showed a different organization of nanocrystals depending on the location inside the ELR matrix, which seemed to be primarily influenced by the angles and confined regions delineated by the surface topographies. For instance, nanocrystals growing inside the 180° angle geometry of the ELR matrix (highlighted by red and green circles in Fig. 4G) were organized co-aligned to each other. However, the 270° angle geometry exhibited more spaced nanocrystals with wider co-alignment angles than planar (180°) and right angle (90°) geometries. We observed that these nanocrystals tended to rotate and grow toward the spaces with more ELR matrix and bundled up into flower-like structures (highlighted by yellow circle in Fig. 4G; Fig. 4I). In contrast, nanocrystals at 90° angle geometry (highlighted by blue circle in Fig. 4G) exhibited completely different behavior, growing and populating compactly with much smaller co-alignment angles. These differences in the organization of the nanocrystals depending on the geometry of the matrix (i.e. topographies presenting 90° or 270° angles) further confirm the possibility of using topographies on the surface to guide the growth of the nanocrystals within the bulk.

#### 3.6.2 Physical characterization of nanocrystals

**Fig. 5:**
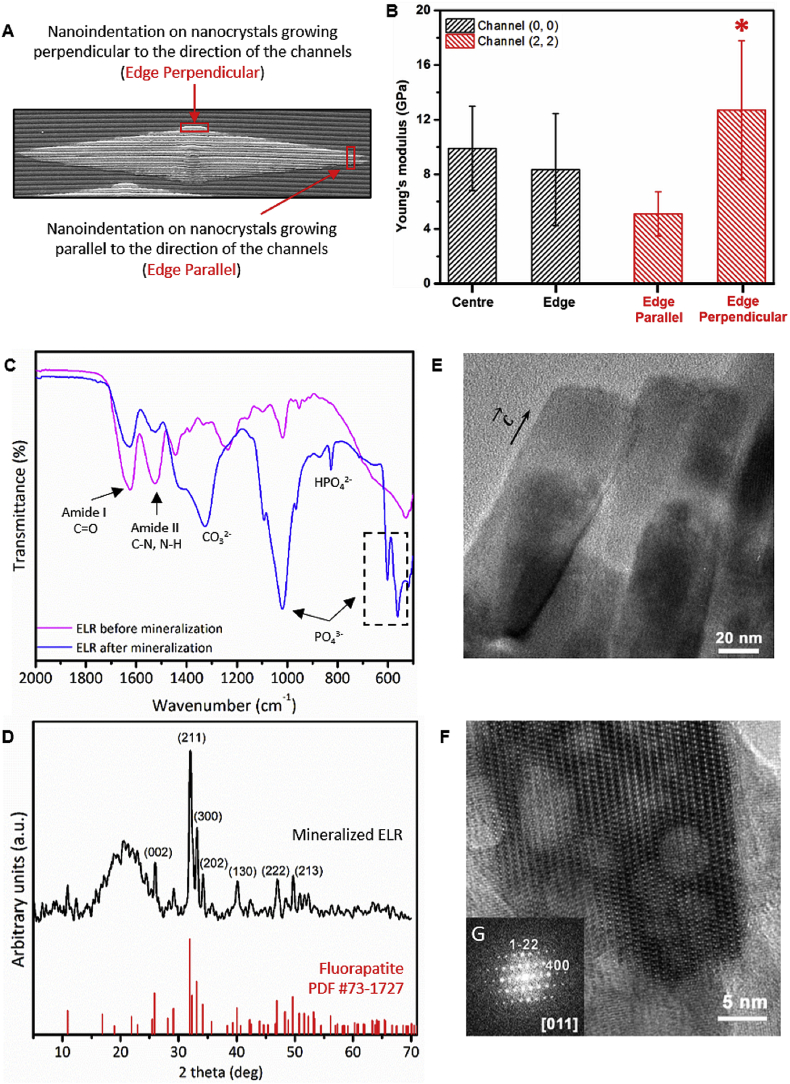
**Physical characterization of the mineralized membranes.(A)** Illustration depicting regions where nanoindentation tests were conducted on the edges of the elliptical-shaped mineralized structures grown on Channel (2, 2) including in the direction parallel and perpendicular to the direction of the channels. (**B)** Graph summarizing the Young's modulus (*E*) measurements at different locations on the circular- and elliptical-shaped mineralized structures grown on Channel (0, 0) and Channel (2, 2), respectively. (**C)** FTIR spectra and (**D)** XRD pattern confirming the presence of apatite structures and the fluorapatite nature of the crystalline phase, respectively, in the mineralized ELR membrane. (**E)** TEM image showing a single nanocrystal of 40–50 nm thick flat-ended geometry and (**F, G)** its corresponding fast Fourier transform (FFT) pattern with typical fluorapatite hexagonal morphology growing along the c-axis. (**B)** ∗ denotes significant difference between *E* values for edge parallel vs. edge perpendicular; *p* < 0.05, estimated using *t*-test in GraphPad Prism software.

Given these differences in nanocrystal organization, we further characterized the mineralized structures and the crystallographic orientation of nanocrystals in both smooth and patterned membranes. First, the mechanical properties of the mineralized structures grown on Channel (0, 0) and Channel (2, 2) were assessed using nanoindentation. Young's modulus (*E*) measurements at the center (9.9 ± 3.1 GPa) and at the edges (8.4 ± 4.1 GPa) of the circular mineralized structures on Channel (0, 0) revealed similar *E* values and were consistent with our previous results [22]. However, for elliptical-shaped mineralized structures on Channel (2, 2), nanocrystals localized at the edges growing perpendicular to the channels exhibited significantly higher *E* values (12.7 ± 5.0 GPa) compared with those on the edges growing parallel to the channels (5.1 ± 1.6 GPa; Fig. 5A and B). We hypothesize that these differences might result from distinct nanocrystal organization in different locations within the ELR matrix, which we explore in the next section. Also, FTIR spectroscopy (Fig. 5C) and XRD (Fig. 5D) analysis of topographically patterned membranes with mineralized structures demonstrated non-stoichiometric apatite spectral peaks that exhibit a crystalline phase and structural parameters that match fluorapatite, respectively, as reported previously by our group [22]. Furthermore, TEM imaging of a single nanocrystal and its corresponding fast Fourier transform (FFT) pattern revealed 40–50 nm thick flat-ended nanocrystals with typical fluorapatite hexagonal morphology growing along the c-axis (Fig. 5E & F). These observations were further confirmed by investigating crystallographic orientation using high-resolution TEM (HRTEM) and SAED. These analyses also exhibited similar fluorapatite characteristics of nanocrystals growing at 90°, 180°, and 270° angle geometries inside the ELR matrix (Fig. 4G). Thus, these results demonstrated that nanocrystals exhibit similar crystallographic characteristics irrespective of the ELR matrix geometry (i.e. topographies presenting 90°, 180°, or 270° angles).

#### 3.6.3 Effect of different geometries on crystal co-alignment

**Fig. 6:**
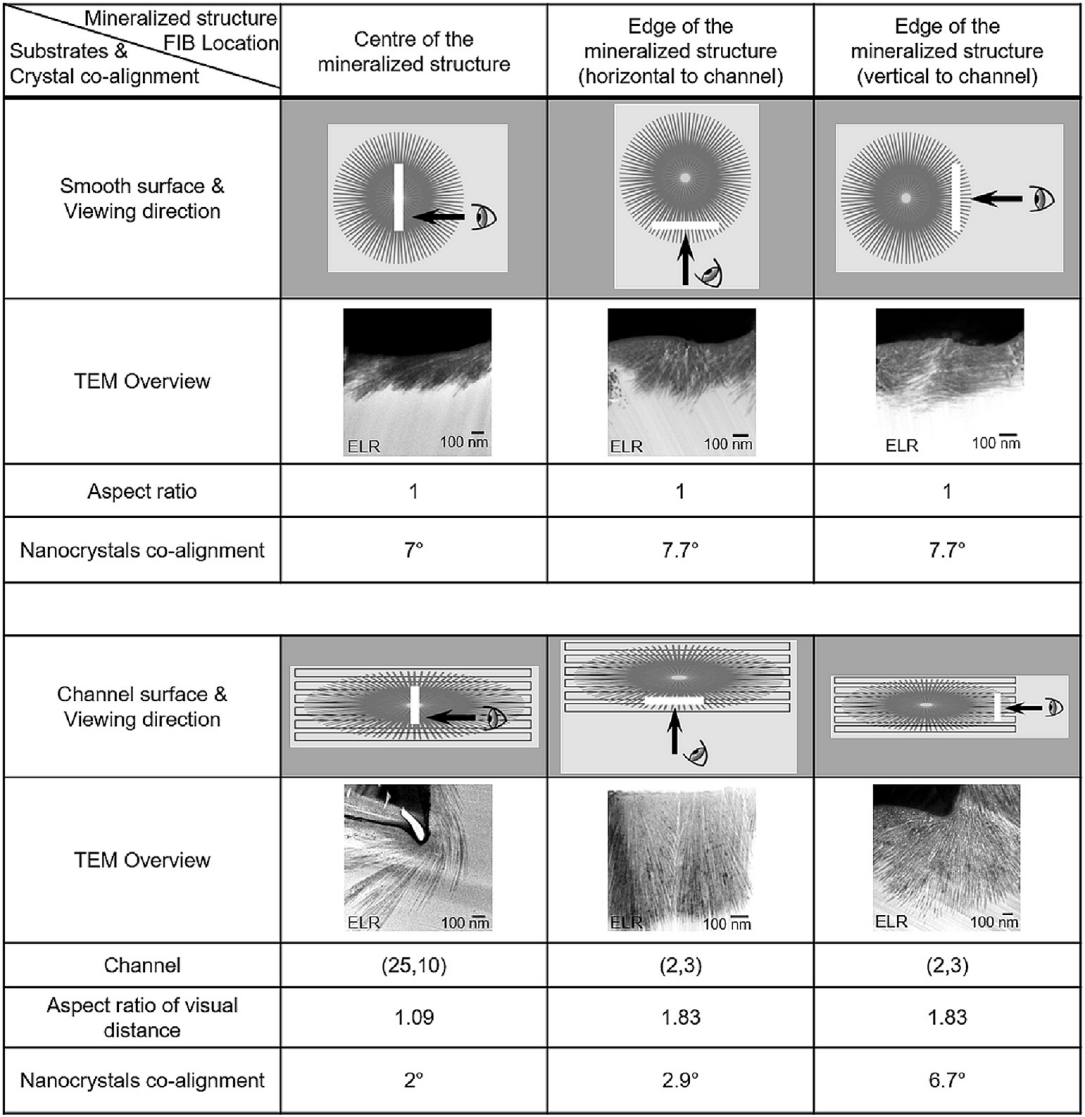
**Co-alignment degree between adjacent nanocrystals.** Table summarizing the relation between aspect ratio and the nanocrystal co-alignment at different locations (i.e. at the center and edges) of the mineralized structures grown on a smooth ELR membrane and on Channel (25, 10) and Channel (2, 3) topographies.

Given these drastic effects of the surface topographies on the growing behavior and morphology of the mineralized structures, we hypothesized that the channel geometry can significantly affect the organization and co-alignment of nanocrystals, which can further regulate the morphology of the mineralized structures. Therefore, we investigated the organization of the nanocrystals growing in Channel (2, 3) at the edges and center of the mineralized structures (Fig. 6). FFT and SAED analysis at these different locations demonstrated that the nanocrystals shared a similar growth orientation (i.e. along the c-axis) but different co-alignment degrees ranging between 2° and 7°. We hypothesized that this considerable difference in nanocrystal co-alignment may play a role in the preferential growth of the nanocrystals along the channels compared with perpendicular to them. We reasoned that differences in nanocrystal co-alignment would have significant effects on the morphology and mechanical properties of the mineralized structures at the macroscale. FFT analysis of nanocrystals at 180° angle geometries revealed nanocrystal co-alignment of 2.9°, whereas at 270° angle geometries, they co-aligned at 6.7° (Supplementary Fig. S3A and Fig. S3B). This greater degree of nanocrystal co-alignment along the ridge or channel direction enables more presence of ELR matrix between nanocrystals. We speculate that this higher availability of ELR matrix between nanocrystals (i.e. separated by an angle of 6.7°) in the direction of the channel compared with those growing perpendicular to the channel (i.e. separated by an angle of 2.9°) would lead to enhance the growth of the nanocrystals as a result of their affinity to the ELR [22] and a higher diffusion of Ca and P ions and local increase in ion concentration. Similar observations have been reported on apatite nanocrystal formation during mineralization of collagen fibrils because of an increased concentration and localization of Ca and P ions within confined gap regions [42,43]. In contrast, nanocrystals exhibiting lower amounts of ELR matrix between them (i.e. 2.9°) would allow less CaP diffusion and consequently more restricted crystal growth. Furthermore, as expected, densely packed nanocrystals because of higher co-alignment (2.9°; i.e. at the edges growing perpendicular to the direction of channels) exhibited higher *E* values (12.7 ± 5.0 GPa) compared with those with lower co-alignment (6.7°; i.e. at the edges growing parallel to the channels; 5.1 ± 1.6 GPa). Thus, these results demonstrate that differences in nanocrystal alignment in the direction perpendicular and parallel to the channels lead to the effects on growth preference and mechanical properties of the mineralized structures at the macroscopic level.

## 4 Conclusion

The present work reports on the possibility to guide mineralization with spatial control by integrating a mineralizing matrix and surface topographies. Apatite nanocrystals nucleated and grew inside of the ELR matrix, whereas topographical patterns on the surface were able to generate ELR volumes with specific geometries, which dramatically affected mineralization. In summary, (1) we demonstrate that minor changes in single nanocrystal co-alignment led to large mineralization effects at the micro and macroscale, (2) we validate the possibility of using our approach to selectively guide the growth of the hierarchically mineralized structures by systematically modifying surface topographies, and (3) our study provides new knowledge on the role of spatial confinement of the organic matrix on the growth of crystals at nanoscale and microscale. In addition, our study addresses a major challenge in materials science by establishing the possibility of controlling various structural properties such as crystal orientation, spatial organization, directional growth, and hierarchical organization over a large millimeter scale. We envisage the possibility of developing biomineralizing synthetic materials with advanced functionalities that can offer exciting opportunities for a broad range of fields expanding from materials science to hard tissue (such as dental enamel and bone) regeneration.

## Data and materials availability

All data are provided in the article or the supplementary file.

## CRediT author contribution statement

This study was conceptualized by X.D., S.E., E.T.-M., A.H., and A.M. X.D. and A.H. performed the experiments. PDMS masters using photolithography was prepared by J.M.S.-D., N.C., and N.G. TEM was performed and analyzed by N.V.T. and E.N. G.G. and N.M.P. performed and analyzed the mechanical properties. A.B. provided support with the characterization of the materials and correction of the article. J.C.R.-C. provided support with the synthesis of ELP molecules. A.H. and A.M. wrote and revised the article. M.A.-J. revised and corrected the article. A.M. supervised the work.

## Declaration of competing interest

The authors declare that they have no known competing financial interests or personal relationships that could have appeared to influence the work reported in this paper.
