# Supplementary material for: Topographically guided hierarchical mineralization
Source: Mater Today Bio. 2021 Jun 9;11:100119. doi: 10.1016/j.mtbio.2021.100119 (PMC8273417; doi:10.1016/j.mtbio.2021.100119)
Supplement: Multimedia component 1 [file mmc1.docx]

**Topographically guided hierarchical mineralization**

Xinru Deng^1,2,Ψ^, Abshar Hasan^3,4,5,Ψ^, Sherif Elsharkawy^6^, EstherTejeda-Montes^7^, Nadezda V. Tarakina^8^, Gabriele Greco^9^, Elizaveta Nikulina^10^, John M. Stormonth-Darling^11^, Neil Convery^11^, Jose Carlos Rodriguez-Cabello^12^, Alan Boyde^13^, Nikolaj Gadegaard^11^, Nicola M. Pugno^1,9^, Maisoon Al-Jawad^14^, Alvaro Mata^1,2,3,4,5,^*****

^1^School of Engineering and Materials Science, Queen Mary University of London, E1 4NS London, UK.

^2^Institute of Bioengineering, Queen Mary University of London, E1 4NS London, UK.

^3^School of Pharmacy, University of Nottingham, NG7 2RD Nottingham, UK.

^4^Department of Chemical and Environmental Engineering, University of Nottingham, NG7 2RD Nottingham, UK.

^5^Biodiscovery Institute, University of Nottingham, NG7 2RD Nottingham, UK.

^6^Faculty of Dentistry, Oral & Craniofacial Sciences, King's College London, London SE1 9RT, UK.

^7^Mabxience, Insud Pharma, León, Spain.

^8^Max Planck Institute of Colloids and Interfaces, Potsdam-Golm Science Park, Am Mühlenberg 1 OT Golm, 14476 Potsdam, Germany.

^9^Laboratory of Bio-Inspired, Bionic, Nano, Meta, Materials & Mechanics, Department of Civil, Environmental and Mechanical Engineering, University of Trento, 38122, Trento, Italy.

^10^CIC Nanogune, Tolosa Hiribidea, 76, E-20018 Donostia – San Sebastian, Spain.

^11^James Watt School of Engineering, University of Glasgow, G12 8QQ Glasgow, UK.

^12^BIOFORGE Group, University of Valladolid, CIBER-BBN, Valladolid, Spain.

^13^Oral Bioengineering, Queen Mary University of London, E1 4NS London, UK.

^14^School of Dentistry, University of Leeds, LS2 9JT Leeds, UK

**^Ψ^** Equal contribution

* Corresponding author: [a.mata@nottingham.ac.uk](mailto:a.mata@nottingham.ac.uk)


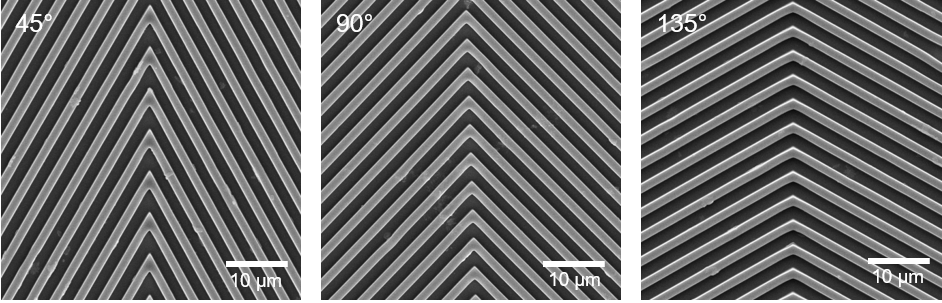


**Figure S1.** SEM images showing surface topographies of zig-zag channel (2, 2) with varying corner angles including 45°, 90°, and 135° before mineralization

**
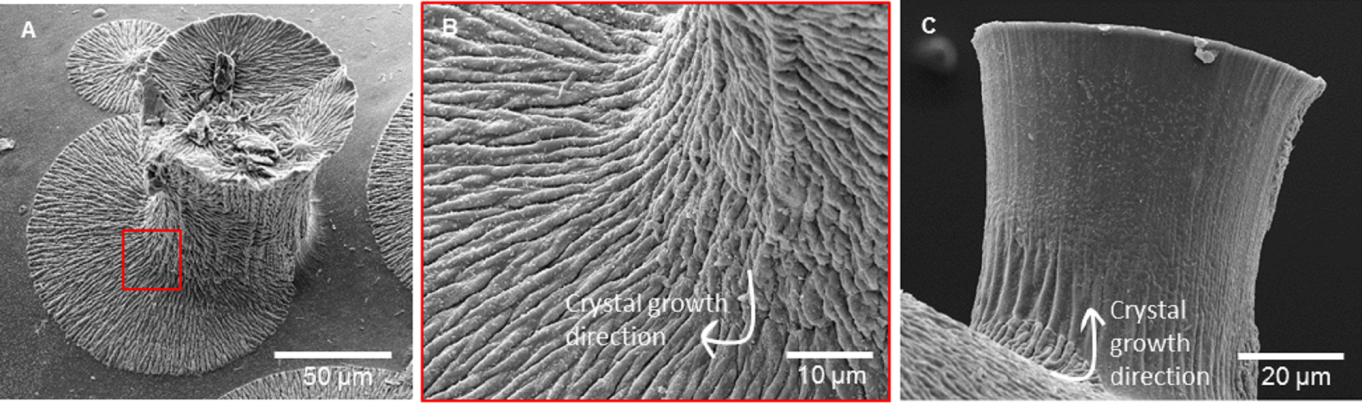
**

**Figure S2.** SEM images showing (A) crystal growth direction from the vertical wall of the post to the horizontal space between posts, (B) zoomed-in image from A, and (C) crystal growth direction from the horizontal space between posts to the vertical wall of the post.


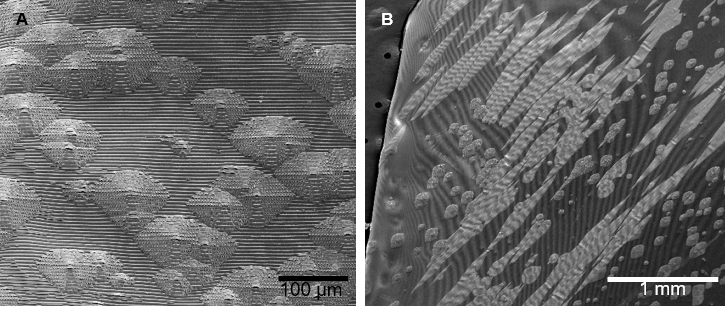


**Figure S3.** SEM image showing mineralized structures on **A** Channel (1, 2) and **B** Channel (2, 2).


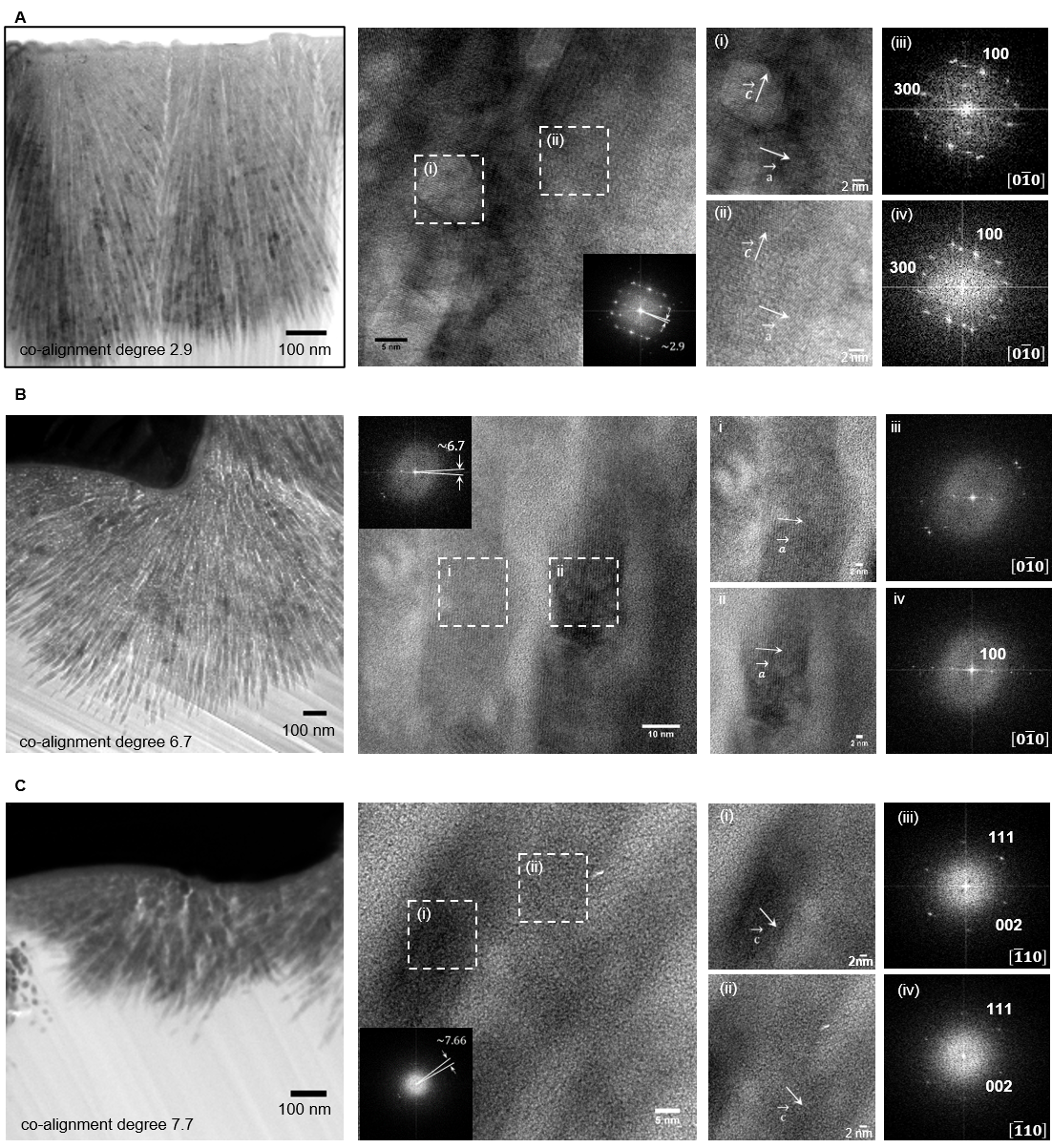


**Figure S4.** TEM image showing nanocrystals organization in the FIB milled-out lamella from the edges of the mineralized structure grown on Channel (2, 3). **(i), (ii)** HRTEM images of the single fluorapatite nanocrystals showing the growth orientation and crystal lattice with their **(iii), (iv)** corresponding FFT analyses **A** showing 2.9° co-alignment angle between nanocrystals at 180° angle geometry and **B** 6.7° co-alignment angle at 270° angle geometry. **C** HRTEM and FFT analyses of the single nanocrystals milled-out by FIB sectioning from spherical mineralized structure grown on smooth membrane showing nanocrystals co-aligned at 7.7°.
